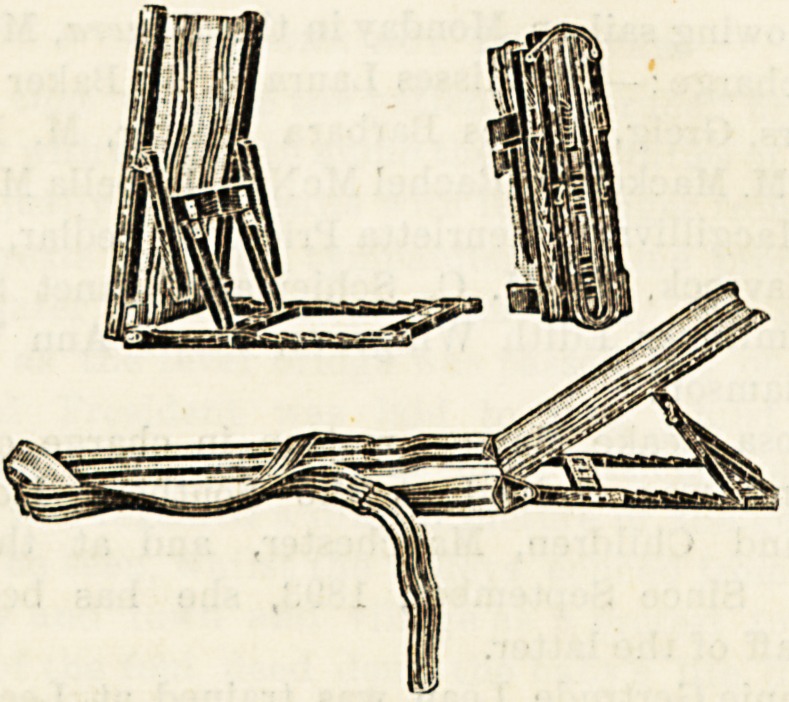# The Hospital. Nursing Section

**Published:** 1902-02-01

**Authors:** 


### The Hospital.

### Nursing Section.

Contributions for this Section of "THE HOSPITAL" should be addressed to the EDITOR, "THE HOSPITAL" NURSING SECTION, 28 & 29 Southampton Street, Strand, London, W.C.

No. 801.—VOL. XXXI. *SATURDAY, FEBRUARY* 1, 1902.

### Notes on News from the Nursing World.

### THE ROYAL BRITISH NURSES' ASSOCIATION.

QUEEN ALEXANDRA has graciously given her patronage, together with Princess Henry of Battenberg and the Duchess of Connaught, to the sale which is to be held under the presidency of Princess Christian, at Lord Brassey's house, 24 Park Lane, on February 6th and 7th. The sale will be opened on February 6th, at 2.30 P.M., by Princess Christian, who will assist at one of the stalls during the afternoon, and on February 7th at the same hour by the Marchioness of Lansdowne. As already announced, the proceeds will be devoted to the fund for building a nurses' settlement, in which nurses disabled in the pursuit of their calling or retired from work may have free quarters and enjoy the comfort and independence of home life without the heavy strain on their savings of existing high rents. The stalls will present unusual attractions, many handsome collections having been sent from Egypt and India. There are also many interesting war relics and other objects from South Africa, rare stamps from Syria, several beautiful specimens of art needlework and a fine collection of pictures both in oil and water-colours for sale. Princess Christian's stall will consist of leather and bric-à-brac. St. John's House, Norfolk Street, Strand, and the London Association of Nurses, will each have a separate stall, the desire in both cases being to raise sufficient money to endow quarters in perpetuity for one of the staff.

### "B.-P" AND THE NURSES' CLUB.

GENERAL BADEN-POWELL writes to us from the South African Constabulary, Zuurfontein, Transvaal, under date of December 18th, 1901, that some months ago "a club of nurses somewhere in England" wrote to tell him that they had given his name to their club. He continues: "I wrote in reply acknowledging the honour done to me, but that letter never reached them, as it has lately been returned to me, having been recaptured from some Boers who had stolen it from our mails." General Baden-Powell adds that, having forgotten the address of the nurses' club to which it was addressed, he has been unable to send it on to them, and asks if we can help him in the matter. We have no doubt that some member of the club to which he refers will see this and will be pleased to forward to us the full address in order that the General may be able to transmit his reply a second time. It is not likely to be again stolen by the Boers from our mails.

### THE LOCAL GOVERNMENT BOARD COMMITTEE.

IT is, of course, ridiculous to blame the President of the Local Government Board because he did not nominate a woman on the committee which he has appointed to inquire into the subject of the nursing of the sick poor in workhouse infirmaries. A departmental committee must, in the nature of things, consist of members of the department only. Women, whether matrons or guardians of the poor, can render better service to the movement for the reform of nursing by offering to give evidence before the committee than by sitting upon it. The constitution of the committee is a guarantee that the statements of all classes of persons qualified to afford information on the points under consideration will be welcomed.

### THE WAR NURSES.

THE *Mohawk* arrived from South Africa on Saturday last, the following nursing sisters being on board:—M. S. Milne, requires three weeks' leave, and returns to South Africa; F. H. Barry, wishes to return to South Africa when required; E. Johnson, wishes to return to South Africa when required. All A.N.S.R. The *Roslin Castle*, which is due at Southampton February 13th, has on board Nursing Sisters A. M. Harrison, C. Harris, E. C. O. Leggatt, K. E. Nisbet, and T. H. Jones.

### PADDINGTON AND BERMUDA.

IF only the other London boroughs would support the Women's Memorial to Queen Victoria as handsomely as Paddington has done the central fund would be in a flourishing condition. At a meeting of the Paddington branch, which was held at the residence of Sir John Aird, M.P., mayor of the borough, it was announced that the amount subscribed had reached £1,400, and that the list was now closed. This is the largest contribution from any London borough, but the fact is due, not only to the liberal manner in which the residents have responded to the appeal, but also to the generosity of the mayor, who has paid all the expenses of collection. Thus there will be no deduction in the sum forwarded to the general treasurer. A very gratifying example has also been set by the colony of Bermuda, which has contributed £76 to the movement. The most interesting feature in connection with this result is that the women of every district in the island are represented on the cards of the collectors.

### THE BRISTOL GUARDIANS EQUALLY DIVIDED.

IN spite of the vigorous manner in which several of the Bristol Guardians advocated at the last meeting the extension of the nurses' holidays from a fortnight to three weeks, the motion in favour of the change was not carried, 27 of the board voting each way. But this means that the concession will be made. The opponents to it are far more likely to diminish than to increase. We rejoice that the ladies on the board, one of them bearing the honoured name of Nightingale, strongly urged the extension on the grounds of consideration for the nurses and of real economy. It was pointed out that last year a sum of between £70 and £80 was spent in advertising, and, in fact, the only plea in defence of the maintenance of the existing order of things was that "other unions have the same trouble as the Bristol Guardians in obtaining nurses." This, no doubt, is true, but do the Bristol Guardians, then, desire to remain in the category of public bodies who are behind the times? We hope that at the next opportunity they will determine that the ancient city shall be free from such a reproach. It is with pleasure we note that the *British Medical Journal* in an article on the general question supports our view, and affirms that "Nurses should certainly have three weeks' vacation so as to get a thorough change."

### FRICTION AT BARNSLEY.

The Barnsley Board of Guardians, instead of quietly accepting the resignation of the superintendent nurse and another of the staff, wisely decided to hear from them why they had resigned. The superintendent nurse gave a very sufficient, though not from the point of view of the Guardians a very satisfactory, reason. She said that she had resigned "because she had got a better appointment, and because she had grown tired of workhouses." The other nurse entered into details and made several complaints respecting the manner in which she had been treated by the workhouse matron. The latter being summoned to appear, the nurse repeated the complaints in her presence, and the workhouse matron declared that they were "quite new" to her. One of her answers strikes us as singularly weak. Concerning the nurse's allegation that she did not get things when she asked for them, the workhouse matron rejoined that if that were so "it was because the articles had been taken into the wrong ward." Then the workhouse master chimed in and remarked that "everything a nurse asked for was not supplied, because they had to check useless extravagance." In other words, it rests with "they," the untrained officials at the Barnsley workhouse, to decide whether or not the trained nurses shall receive for their patients the things they require. It is not surprising that there is the "constant changing of nurses" to which the Guardian who suggested the attendance of the retiring superintendent and her colleague referred.

### A "QUEEN'S NURSE" SUBSCRIBER.

In a colliery district in South Wales a "Queen's Nurse" is kept whose salary has lately been raised by means of public subscription. Amongst the subscribers was one old lady who, having paid her promised sum two years running, declined on being asked the third time to give any more as she said that she had not required the services of the nurse. Shortly afterwards she went to visit a neighbour who had been very ill, and had greatly benefited from the nurse's visits. On hearing from her friend how helpful the nurse had been, the old lady said she would change her mind and pay her subscription as before, "for there," she added, "a nurse is 'andy after all."

### APPRECIATING A DISTRICT NURSE.

At the annual meeting of the subscribers to the St. Nicholas Parish Nursing Fund, Newbury, the treasurer announced that the receipts for the past year had been £104, and that a balance was left in hand of £12 19s. 5d. The nurse treated 430 patients during the year, and paid 4,045 visits. Of these 430 patients, 237 had been cured, 67 were chronic cases, and 29 had died. These figures pointed to the extensive nature of the nurse's work, and that there was no distinction of creed was evidenced by the fact that of the nurses' patients 340 were Church of England, 87 Nonconformists, two Roman Catholics, and one "nothing." Dr. John Watson spoke in appreciation of the good done by the nurse, who had been working in the parish for ten years, and he seconded the proposal that her salary should, as a substantial expression of the gratitude of the town, be raised from £90 to £100. As a medical man he assured his hearers that the nurse did her work thoroughly and conscientiously, the visits not merely consisting of running in and out of a house, but frequently as he knew, entailing over an hour's work. If the funds would allow he would like to see her salary raised to £200, and even if they gave her £500 it would not be too much, a remark which was followed by laughter and applause. The resolution to raise the salary to £100 was then cordially adopted. It was decided to make an endeavour to increase the number of subscribers, and it was hoped that collections might be made at some of the chapels.

### THE EPIDEMICS AT COVENTRY.

For the first time since the commencement of their work 16 years ago, the Coventry Nursing Association had to announce a deficit in their financial report at the annual meeting this year. The expenditure exceeded the receipts by £92 8s. 10d. This, the Managing Committee believed, was almost entirely owing to the great drain on their resources brought about by the serious amount of illness which was prevalent in Coventry last year. Early in the spring the flood brought sickness in its train, which was followed by an outbreak of typhoid and later by an epidemic of scarlet fever. Not only did this involve a heavy call on the funds of the association, but it sorely taxed the strength of the nurses. In consequence of the strain imposed by the increase of work, one of the nurses was obliged to give up her duties and take a thorough rest in Scotland, and she is remaining away a further three months in order that her health may be quite re-established. The pressure at one time was so great that it was found necessary to engage two nurses from the Warneford Hospital, which, of course, added to the already heavy expenses. There is now a staff of six nurses, who, during 1901, paid 22,379 visits. With regard to the funds, the joint secretary has written to several of the large manufacturing establishments which did not contribute, and asked for subscriptions. In many cases the replies have been satisfactory, in others the answers have yet to come. It was proposed at the annual meeting to make a special appeal to the public for assistance, and to endeavour to get the Guardians to increase their subscription of £12 per annum in view of the work done by the nurses for the poor. One gentleman doubled his subscription to the institution before he left the meeting.

### ANOTHER CHANGE AT NEWTON ABBOT.

The Newton Abbot Guardians have now to fill the vacancy occasioned by the resignation of the superintendent nurse. Miss Fisher must have had a trying time since she has been at Newton, where, though the new infirmary is one of the best in Devonshire, the constant changes of nurses have rendered the work exceedingly difficult. She has obtained another appointment, and the Guardians, in accepting her resignation, tendered her their thanks for the manner in which she had discharged her duties, while the house committee expressed regret at the loss of her valuable services.

### A MODEL BRANCH.

A STRIKING record of work by one of the Queen's nurses was cited at the first annual meeting of the Totnes Benefit Nursing Association. The committee reported that from February 1st to December 31st, she made no less than 3,502 visits to 105 members and 23 special fee patients. "When the nurse came she saw and conquered," said the president, and "in my presence she has been called a ministering angel." Miss Peter, the superintendent of Jubilee Institute nurses, on the occasion of her visit to the town found that the organisation was splendid, and said she should report Totnes as a model branch.

### NURSES' NEEDLEWORK GUILD.

THE fifth annual report of the Nurses' Needlework Guild shows that during the year 1901 sixteen new members joined and seven resigned. There are now 308 members and associates' names on the books. The annual sale of work was again held at St. Andrew's House, Mortimer Street, by the kind permission of Miss Debenham. There were nearly 500 garments sent in, 13 more than in 1900, but the committee have again to report that many members failed to comply with the conditions of membership and did not contribute anything. On the other hand, one lady made 60 garments and the others 20 each. Cordial letters of thanks from the different hospitals to which parcels of articles were forwarded have been received by Miss Theobald, the honorary secretary and treasurer of the Guild.

### SHEFFIELD DISTRICT NURSES.

THE report for the year ending September, 1901, of the work done by St. George's Home for District and Private Nursing, Sheffield, shows that 966 cases were nursed, 19,767 visits were paid, and 117 patients died. While the number of cases has again diminished, the number of visits has increased considerably. This is accounted for by many patients having to be visited both morning and evening, as in cases of typhoid, for several weeks. The need of nurses has never been more urgent, but instead of being able to add another nurse to the number now at work, the year closes with a deficit of over £8. The City Council have again contributed their much-appreciated gifts of tramway tickets, and the welcome cab has, through the kindness of friends, been continued. There has only been one change in the staff during the year, the "Wicker" nurse having left to be married.

### A NURSE REPRESENTED ON A REREDOS.

IT is not long since we recorded the appearance of the figure of a nurse in a church window designed as a memorial to some of the victims of the war in South Africa. Now we learn that the new reredos which has been designed for a church at Bristol, as a memorial of the work of Archdeacon Wilson, contains, besides the figures of the Commander-in-Chief and the Bishop of Bristol, that of a nurse.

### A POSSIBLE OPENING.

IT having been suggested that nurses who, from physical causes such as an incurable injury to a knee or an ankle, have been compelled to give up nursing, might be glad to obtain employment as head sempstresses at the hospitals of the Metropolitan Asylums Board, we asked the Clerk to the Board to inform us whether such persons are eligible for the appointments. He informs us that applications from them "provided they are qualified by experience, would be considered with others," and he adds that "persons appointed to such posts are subject to satisfactory medical evidence of physical fitness before being permitted to take office." These conditions are, of course, essential; but a nurse might be both qualified by experience to act as sempstress and be physically fit to perform the duties. As the salaries in future of head sempstresses at the fever hospitals of the board are to be £30 a year, and at the small-pox hospitals £32, with board, lodging, washing, and uniform, the position seems likely to offer an opening to nurses who, from no failure of general health, have been compelled to give up nursing.

### PROGRESS AT LICHFIELD.

AT the annual meeting of the subscribers to the Lichfield Victoria Nursing Home it was announced that the sphere of usefulness of the institution had been much increased during the past financial year. Thirteen patients were treated in the beds of the home, staying there an aggregate of 148 days and nights, as compared with seven and an aggregate stay of 102 days in the previous year. The older work of the Nursing Home, visiting and attending the sick poor in their own homes, has also been continued, and 3,627 visits were made during the year, while 313 supplies of food were issued from the invalid kitchen. The balance in hand is £148, which is however £18 less than that of the year before, and in order to prevent a further drain being made upon the balance, it was determined to appeal for more contributions. The hearty thanks of the committee to the nurses who have worked and are working for the institution were duly recorded.

### A USEFUL SANATORIUM.

THE Royal West of England Sanatorium at Weston-super-Mare, of which Miss Edith Mawe has been in charge for seven years, is now closed until the end of March for repairs and enlarged sanitation. Under Miss Mawe's auspices it has developed to a remarkable extent, the number of patients having increased from 600 to 2,125 a year, many hundreds being also refused for want of room. It is pleasant to learn that there are no financial anxieties, and that the cost of effecting the improvements, about £850, can be met.

### MANSFIELD DISTRICT ASSOCIATION.

DURING the year ending September 30th, 1901, the nurses of the Mansfield and Mansfield Woodhouse District Association, paid 7,645 visits to 349 cases, 42 of these being cases for which a charge was made. The annual dance last month, in aid of the Association was highly successful, the sum of £26 18s. 6d. being realised after payment of all expenses.

### SHORT ITEMS.

MISS SPECK, the matron of Chichester Infirmary, has been granted six months' leave of absence by the governors owing to ill-health. It is hoped that after this rest and a complete change of surroundings she will be quite able to resume her duties.

### Lectures on Gynæcology for Nurses.

BY ROBERT JARDINE, M.D., M.R.C.S., F.F.P. and S.G., F.R.S.E., Senior Physician to the Glasgow Maternity Hospital' Examiner in Midwifery to the University of Glasgow.

### LECTURE III—EXAMINATION OF A PATIENT.

WHERE it is possible a patient should be prepared for examination beforehand. Her bowels should be thoroughly cleared by purgatives or an enema, and the bladder should be empty. If she is to have an anæsthetic she should not have had any solid food for several hours. The patient should wear a loose night-dress, and she may have on her stockings if the legs and feet are to be exposed.

*External Examination of the Abdomen.*—The patient lies on her back with her knees drawn up, or the legs may be left extended. The clothing must be so arranged as to expose the abdomen from the epigastrium to the mons veneris. The whole abdomen can thus be inspected, palpated, percussed, or auscultated.

*Inspection of the External Genitals.*—This is only necessary when the patient complains of tenderness, or of something protruding there, or if venereal disease is suspected. In diagnosing pregnancy it is also sometimes of importance to inspect the vulva and vagina for the bluish discoloration. The external genitals may be inspected with the patient lying on her left side, with the knees well drawn up, but you get a much better view of them with the patient on her back, and the thighs separated, and well drawn up. This is easily arranged by laying two sheets, or two bath-towels, edge to edge, but overlapping a few inches down the front of the patient, fixing them firmly together by a strong safety pin just over the pubes, and then tucking them in separately round the inner side of each thigh and leg. By this means all exposure, except of the parts actually to be examined, is avoided.

*Vaginal Examination.*—This, as a rule, should not be made at all in young girls, and in unmarried women only when absolutely necessary. It is always the nurse's duty to ascertain whether the "period" has come on, which sometimes happens unexpectedly from nervous causes, and if this should be the case, to inform the doctor quietly of the fact, before any proceedings have commenced, when he will postpone the examination unless there is some urgent reason to the contrary. There are special couches and gynæcological chairs for examining patients on, but an ordinary couch or bed does quite well. The patient may lie in the ordinary obstetric position, *i.e.*, on her left side, with the thighs well drawn up, the right one a little higher than the left, or in Sims' position, which only differs from the above in that her left arm is brought behind her so that the left side of the chest is on the bed rather than the left shoulder. The legs and body are covered, and only the buttocks exposed. The nurse should see that the external genitals have been thoroughly cleansed.

*Bimanual Examination.*—This is the most important of all the examinations. The patient should lie on her back with the knees well drawn up and the feet well apart. On a proper couch or chair the legs would be held up by supports. The legs may be covered separately, if necessary, to prevent chilling of the patient. Both hands are used, one over the abdomen, and the other with one or two fingers in the vagina. The patient should be encouraged to relax her abdominal wall as much as possible, by getting her to breathe deeply or by engaging her in conversation.

*Recto-Vaginal Bimanual.*—This is done in exactly the same way, only the middle finger is passed into the rectum, and the fore finger into the vagina.

*Rectal-Bimanual.*—In unmarried women or young girls the bimanual examination may be made by passing the internal finger into the rectum only. If a further examination is to be made with a speculum, sound, etc., the nurse must see that the instruments are ready sterilised and soaking in a warm solution of lysol (1 in 100). She must also have ready pledgets of sterilised wool, swab holders and forceps.

In all examinations undue exposure of the patient must be avoided. It is difficult to exaggerate the feelings of many sensitive women under the ordeal of examination, and no pains should be spared to lessen the trial. To you it may be merely a case, but do not let the woman feel this. At the same time no mock modesty must be allowed to interfere with the observance of every aseptic precaution.

We must now briefly consider normal menstruation. This being a function in regard to which patients will often confide to nurses details which they hesitate to mention to the doctor, it is essential that the nurse should be able to appreciate the meaning of those divergencies from the possible normal which so often occur.

In temperate climates like our own menstruation usually begins about the age of 14; in hot climates, as in India, it begins much earlier, while in the arctic regions it is much later. In health it should recur at more or less regular intervals, except during pregnancy or suckling, until about the age of 45, when it ceases, the time of its cessation being known as the "change of life," or menopause. Both at the commencement and towards the end of its continuance the periods are apt to recur irregularly. At both of these times the nervous system is often in a very irritable condition.

The periods usually recur every 28 days, but in some the time may be every three weeks. In the normal condition they last from three to six days, but they may in some cases be longer or shorter. Just before the period begins there is a flux of blood towards the pelvic organs. The ovaries and uterus become hyperæmic—*i.e.*, have more blood in them than usual; the endometrium becomes engorged with blood; the glands in it secrete actively; the membrane then breaks down, and by the end of the period the endometrium is practically all shed. In some cases it comes away in shreds, or a complete cast of the uterine cavity may be passed, which, however, must be regarded as abnormal. The endometrium is quickly re-formed.

During the period a woman usually suffers more or less discomfort in the pelvic region. It may only be a feeling of heaviness, but often there is considerable pain, especially if clots are passed, which, again, must be regarded as abnormal, and should be reported. The general health will in many cases be affected. Headache and backache are very common. There may be uneasiness, or even pain, felt in the breasts. During all this time women should take special care of themselves. If women would only bear this in mind many of them would be saved a good deal of suffering, and nurses may render good service by impressing this upon them.

There are different theories about the causation of this periodic function, but they need not be here discussed. It should be understood, however, that the notion that it is only at these times that ova are discharged from the ovaries is now known to be an error.

### Wants and Workers.

WILL anyone having a bed-rest, table, or old linen, etc., kindly let Nurse Ireby, Mealsgate, Carlisle, have them for the use of her poor patients in the district? The people are in need of so much and there is so little.

### Beyond the Seas: Nursing at Port Royal, Jamaica.

By an Occasional Correspondent.

The little settlement of Port Royal, in Jamaica, commands the entrance to Kingston Harbour. It is a naval station, and has a very fine hospital. The patients are all men, chiefly sailors and dockyard employés, but as they have no hospital accommodation for the detachment of engineers and artillery stationed there, the soldiers are admitted on payment of a small sum by the military authorities. The hospital is a two-storied stone building, with wide verandahs facing the sea, with room for 200 patients. The wards for both officers and men are in the upper storey, the lower one being occupied by a large dispensary, the surgeon's office, out-patient rooms, and medical store rooms, a considerable quantity of drugs, etc., being stored here for the use of the ships in the North American and West Indian squadron.

Colourrd Male Nurses.

The staff consists of a deputy inspector-general, two surgeons, and four permanent ward nurses (coloured men) who are trained by the surgeons. As the wards are seldom full, and the surgeons do the dressings, etc., the small number of nurses is sufficient; in cases of emergency they are reinforced by nurses from Kingston. The convalescent patients do the sweeping, dusting, etc., while the scrubbing and lavatories are done by the hospital labourers. A separate block is being built for infectious cases, which have hitherto been nursed in a separate ward in the main building.

Jack as a Patient.

The cases are chiefly malarial fever, aggravated by alcoholism, with an occasional accident from the dockyard. Jack makes a very amusing patient. The luxuries of hospital life ashore, very different from the cramped sick quarters on board, prove too fascinating, and all sorts of dodges are resorted to to get into hospital. I think this old service yarn will bear repeating. Naval surgeon to A.B. seaman, who has come in to be examined for admission. "Well, my man, what's the matter, you don't look very ill?" "Well, sir, it's like this; I eats well, and I sleeps well, but when I gets a job of work I am all of a tremble!" On Christmas Day a burly blue-jacket turned up in the out-patient room, with a frightful black eye, the result evidently of too much Christmas grog. "Please, sir, was the explanation, "I walked into a broom handle by mistake." History is silent as to who was at the other end of the broom. At the beginning of last year an epidemic of yellow fever broke out in the garrison. The troops were promptly ordered to the hills, but not before several deaths occurred, and from January till July fresh cases were cropping up in the most unexpected places.

Private Nursing.

I would not recommend Jamaica, nor indeed any of the West Indian islands as a field for private nursing enterprise, unless the nurse has private means. That nurses are badly wanted there is no doubt, especially for the military and naval stations; but the islands are all suffering from financial depression, and people are seldom able, or willing, to pay one or two guineas a week for private nurses. The cost of living too is great. Servants with the most elementary ideas of their duties, ask, and get from 6s. to 8s. a week. Washing is inexpensive on the whole, but very badly done. Fruit and vegetables are both plentiful and cheap, but fresh milk is almost unobtainable, and fresh butter as bad. A limited quantity of the latter is imported once a fortnight by the Royal Mail and is retailed at 2s. a pound. Mutton can only be got once a week at 1s. a pound, beef is cheaper, 6d. a pound, and can be got every day, but it is very tough and invariably rendered more so in the cooking. Eggs sell from 1d. to 2d. each according to the time of year, and are very uncertain in quality. Clothing is very dear, and if imported a duty of 16 per cent. is charged.

Climate and Scenery.

The scenery is magnificent, but then, as a man remarked to me once, "You cannot get fed up on scenery and nothing else." I spent a few days with some friends, in a bungalow in the hills, about 4,500 feet high. I drove the greater part of the way and then rode the last few miles on a mule. The hills on both sides of the rugged mountain path were a gorgeous mass of pink begonias and gold and silver ferns, but every scrap of food had to be fetched from the plains. The climate at Clifton Mount was delightful in the summer months; quite like an English spring without the east winds, but in winter the damp is excessive. Port Royal is the least picturesque part of the island, but it has a weird fascination of its own. It is nothing but a long narrow sandbank; in some places the soil is only 18 inches deep, and at high tide the hospital verandah on the ground floor gets quite damp. Here is the Naval Dockyard, the Garrison Forts, and the Naval Hospital, all that is left of the old town, which disappeared 200 years ago beneath the waves. A buoy just opposite the hospital, marks the spot where the old cathedral once stood, and on clear days, people say the ruins are still discernible.

A Desolate Cemetery.

I think my greatest bugbear was the naval cemetery. How I dreaded being buried there. It was situated about the middle of the Palisadoes, a narrow sandbank, nine miles long, connecting Port Royal and Kingston. It was a most desolate, lonesome place, the sea on both sides, and full of land crabs. I am thankful to say that they have a better one now on the mainland the other side of the bay. However, in spite of all the discomforts and dangers of yellow fever, etc., I spent two very happy years at Port Royal—enjoyed very good health too. As we steamed out of the harbour, and the guardship H.M.S. *Urgent* dipped her flag, and signalled farewell to us, I said good-bye to Jamaica, with a sorrowful feeling that I should never see her lovely shores again.

### Where to Go.

Savoy Hotel, February 10th and 11th.—Entertainment on behalf of the new Nursing Home at Charing Cross Hospital.

### To Nurses.

**We invite contributions from any of our readers, and shall be glad to pay for "Notes on News from the Nursing World," or for articles describing nursing experiences, or dealing with any nursing question from an original point of view. The minimum payment for contributions is 5s., but we welcome interesting contributions of a column, or a page, in length. It may be added that notices of appointments, entertainments, presentations, and deaths are not paid for, but that we are always glad to receive them. All rejected manuscripts are returned in due course, and all payments for manuscripts used are made as early as possible after the beginning of each quarter.**

### Sick-Room Cookery.

By Maude Mason, Principal of the Bradford School of Cookery.

### FISH COOKERY.

(*Continued from page* 229.)

Fish is often said to be a "brain food"—it has probably obtained this distinction on account of the ease with which it is digested by people who spend a great deal of time over books, and whose digestive organs are often not very robust. If this food is to be a food particularly suitable for an invalid, the most digestible kinds must be chosen. Generally speaking, fish contains about one-third less albuminates than animal food, and there is considerable difference in the composition of the varieties of fish. On looking at any table of the composition of the several kinds, it will be noticed that the chief difference lies in the amount of fat the fish contains. This determines its degree of digestibility; for instance, salmon contains 7 per cent. fat, herring 8 per cent., and sole only 0·50 per cent. To put it roughly, there are two classes of fish, and to those which contain the oil or fat distributed throughout the flesh, and are often spoken of as "red" or "oily" fish, belong salmon, herring, eel, and mackerel; while to those which have their fat contained in the liver, and which is removed when the fish is cleaned, belong sole, whiting, cod, halibut, turbot, &c., and the latter are generally called "white" fish. From this it will be seen that fish belonging to the "white" group should be chosen for an invalid. Sole is considered the most digestible, then whiting and plaice; salmon, and fish belonging to its group, should never be given to anyone of weak digestion.

### Fried Fish.

After having chosen the fish, let us consider the different ways in which it may be cooked. It may be either boiled, baked, steamed, or fried. Fried fish is only permissible when the patient is well advanced in convalescence; as, however, it is very enjoyable when allowed, I will give a few hints on that way of cooking. Have the fat perfectly clean, do not skimp the amount, see that the fish is fresh. Sole and plaice are generally filleted. After having carefully wiped and dried the fish, dip it first into beaten egg, then have some breadcrumbs on a paper, place the fish in the crumbs and shake the crumbs well over the fish so that every part is thoroughly covered. Now see that the fat is hot enough, and this is really the secret of frying fish well—the fat should have a blue vapour rising from it, and should be perfectly still. Failure sometimes results owing to the common error of speaking of fat boiling, and the fish is put into the fat when it bubbles and is apparently boiling. As a matter of fact it is then at the heat of boiling water, 212° F. any water in the fat is driven off as steam, and causes ebullition, the fat itself goes on getting still hotter, and it is only when it reaches nearly 400° F., the stage at which the blue smoke rises, that it is sufficiently hot for frying. What follows is readily understood—when the prepared fish is placed in the hot fat, the temperature is sufficiently high to at once cook the outside, a firm coating is at once formed, and the juices of the fish cannot escape; the heat of the fat raises the heat of these juices, and thus the fish is actually cooked in its own steam. If the fish is not quite covered with fat it must be turned over and the other side fried. It will be seen that if the fat is not sufficiently hot it soaks into the fish, and the result is a sodden, fatty mass. The piece having been properly fried a nice, golden brown, it is lifted out of the pan, placed on a piece of absorbent paper (the fat should run off like the water from a hard-boiled egg), and then dished up on a dish mat. A little parsley makes a pretty garnish.

### Boiled Fish.

As regards the boiling of fish some cookery books recommend that the fish should be put into cold water and the water brought to the boil; this method, however, naturally draws out the goodness and flavour. The fish should be placed in water just below boiling point with sufficient salt added to make the water taste. The heat of the water acts precisely in the same way as the hot fat in the case of fried fish, the pores immediately become sealed up and the juices prevented from escaping; the salt raises the boiling point of the water slightly, and also increases the density of the water and thus lessens the inclination of the juices to escape. After placing the fish in the water, let the water boil ever so gently for two or three minutes, then draw the pan to one side, and let it barely simmer for the remainder of the time. Allow ten minutes to every pound of fish after it has begun to cook. A little vinegar added to the water makes the flesh firmer.

### In the Oven.

Where a small piece of fish has to be cooked it is even better than boiling it to cook it in the oven in the following way: Butter a tin, place the fish in it, cover with a piece of buttered paper, and cook in a moderate oven. The fish cooks in the steam of its own juices, and a slight amount of liquid from the fish will be discovered in the tin after cooking; this should go into the sauce. Here you get the full nutriment of the fish, and the flavour is most delicate and delicious.

### In a Steamer.

Fish may also be cooked in a steamer over a pan of boiling water. Place the prepared fish in a basin, and cover with buttered paper. The liquid which oozes out should be served with the fish or in the sauce. Any of the three last ways may be appropriately chosen.

### The Sauce.

Now just a word with regard to a sauce. I will cite quite a simple recipe. It should be neither too thick like a paste, nor thin like milk; the consistency of good custard is about right, and, above all, it must be as smooth as velvet. Melt 1 oz. butter in a saucepan, take the pan off the fire, work 1 oz. flour into the butter, then add very gradually at first ½ pint milk only, or ½ pint milk and fish stock (made by boiling any bones of the fish in water). Stir this all the time over the fire till it boils, add salt to taste, and cook a few minutes. The sauce, which can be mixed more smoothly with a wooden than with an iron spoon, may be served plain, or may be used as the foundation of several sauces. It may be turned into parsley sauce by adding finely chopped parsley; the addition of the yolk of an egg and just a very little vinegar makes a nice sauce. Oyster sauce is made by placing to the above quantity about six oysters cut in two with a teaspoon (not a knife), and let the sauce remain on the stove for five minutes without boiling again.

### Oysters.

I must give a word of warning on cooking oysters under any conditions. Raw, they are very digestible; when cooked they are often over-cooked and rendered tough, and therefore indigestible. To avoid this they must never be allowed to get more than warm. Carefully managed there is no reason why they should not be served cooked. The soft part of the oyster is the liver, and the hard part is the muscle which binds the shell together, and should not be given to an invalid either raw or cooked.

(*To be continued.*)

### Lectures to Head Sisters.

By E. Margaret Fox, Matron of Tottenham Hospital, N.

LECTURE IV.—(*Conclusion*).

A patient who has come in for operation in a few days' time needs, of course, very different treatment from you than a bad medical case. Often they are feeling pretty well and able to help themselves, but they are sure to be dreading the coming ordeal, and will need all the bright cheerful words you have time to give them. During the interval before the operation, give them books, needlework, anything to take their thoughts off themselves. Do not let them discuss their ailments more than you can help, and do not make too much of the necessary preparations, for these, especially for an abdominal section, are often more terrifying to the patient than the operation itself. The needful bath can be given as a matter of routine, and if a probationer is entrusted with the preliminary cleansing of the abdomen and preparing it for operation, be there yourself as well, to insure the work being properly done, without the patient's being told it is the nurse's first attempt. Everyone must have a beginning, and some things, like shaving, require a good deal of practice, so, for the patient's comfort, it is well not to let her know the probationer has never done such a thing before. Do all you can to calm your patient's fears and encourage her to be brave, and when the time comes for her to be taken to the theatre allow no delay nor flurry at the last. If possible, give her your special attention for a while after the operation is over. Do not leave her solely to the care of a probationer, but let her see you there as she comes round after the anæsthetic, for she will probably like to know you are beside her. Reassure her about the operation, and try to induce her to sleep by your own calm manner and absence of excitement. Treat everything you may have to do for her as a matter of course, and above all never let her see you are anxious or frightened about her condition. It is not easy, if you have one or two bad cases in your ward, to preserve a quiet, unruffled aspect when you are really feeling very worried about them, and yet you have no right to cast a gloom over those patients who are doing well by your openly expressed anxiety about those who are not. Too much show of feeling on your part will quickly sadden the whole ward.

If a patient is obviously dying, lose no time in putting a screen round the bed, and, if possible, allow his friends to be with him, for they, better understanding his character and wishes than you, can often soothe him by their mere presence. These friends are sometimes very troublesome, and tax your patience and kindliness severely. Still, it must be remembered they are in grief and suffering great anxiety, and so have a large claim on your forbearance.

Should a dying patient express a wish for the chaplain or a minister to visit him, do your utmost to gratify it, but do not press religion unduly on him. If the suggestion of a clerical visit comes from you, it will often frighten the patient, conveying to him the idea that you think all hope of recovery is gone. But here is where your highest and best moral influence will tell, for if day by day you so live in the sight of your patients as to make them see you are working for the life to come and not only for this, then the opportunity is sure to occur for you to be the truest help that one soul can be to another in the direst strait which befalls humanity. But do not try to force the situation or to bewilder them with creeds.

When a patient dies or returns home, either he or his friends may wish to express their gratitude by making you presents or by inviting you to go and visit them. Sometimes it is a little difficult to know how to act, for while you do not want to hurt their feelings by refusing their offers, you do not care to place yourself under any obligation by accepting them. With regard to presents, a safe general rule is to refuse any gifts of a personal nature, but to welcome warmly plants, flowers, or some little article for ward use or ornament, and to encourage them to make donations or become subscribers to the hospital. Expressions of gratitude do not so frequently take a practical form that we can afford to ignore them, for most often, however grateful patients may really be for your kindness, they do not say so. Your reward in serving them really lies in seeing them get well, or in smoothing for them the hard pathway of death.

As to visiting patients after they have left the hospital, if they are very poor, and you are interested in them, of course there is no reason why you should not do so, but when they belong to a better class, and invite you on terms of equality, sometimes one or two objections arise. During their stay in the ward, they have become so familiar with the ways of the hospital, that they naturally ask a good many questions about it, and unless you are more discreet than most, you will find yourself betrayed into retailing to them divers bits of gossip that are better not told to outsiders. They cannot appreciate or understand the peculiar difficulties and environment of our hospital life so well as those who live within its walls, and without understanding, cannot judge it fairly: therefore you may find some harmless little story you have told them much misrepresented, and come back to you in time, with quite a different construction put upon it. In all your dealings with patients, whether in the wards or at their own homes, it is necessary to be most guarded, particularly in what you say about the medical or nursing staff.

Of course you will never let a patient see that you dislike him personally, or that you object to what you have to do for him. If a dressing makes you feel sick, do not say so, nor ask someone else to do for him what you will not do yourself. Cultivate graciousness of speech, for a curt, brusque manner is resented by them as bitterly as it would be by you, and many complaints by patients, when sifted, hark back to some fault of manner on the part of the nurse or other official complained of, rather than the grievance itself. And do not, because you are no longer a probationer, omit to study each case that comes into your ward. If one is especially interesting, write the notes of it in a book of your own, compare it with other similar cases and so enlarge your field of knowledge and experience. To do your duty thoroughly to your patients, it is obvious you must know all you can about their cases, for you can then be so much more of a help to them, and to help each other in the great struggle against sickness and suffering, is after all the highest conception of what your duties are towards your patients and will make you the most sympathetic and efficient nurses.

"Have love. Not love alone for one,
But man as man thy brother call,
And scatter like the circling sun
Thy charities on all."

### New Home at Chester.

Last week the new Nurses' Home in connection with Chester General Infirmary was opened by Lady Lettice Grosvenor. Formerly erected for the reception of infectious cases, the building has been converted, at a cost of about £1,000, into a commodious and well-appointed home for the nursing staff.

### A Baby Typhoid.

By an American Nurse.

Along the telephone line rang a cry for help. From twenty-five miles north of Syracuse it started, and the tremor in the voice of the man who called lost nothing by the annihilation of space, but left the listener holding the receiver very sure that a human heart in sore straits was speaking. Sympathy is a poor factor if unaccompanied by practical proof of its existence, so I called out cheerily: "Will catch that 8-train—meet me—Good-bye," and flew up to my room, where my valise was ready packed for emergency. I only required a rapid change of garment, and flinging adieux to others in the club I passed out, and went on my way to Fulton. *En route* I had time to think of my destination, and to remember what had been told me of the beautifully-situated village with its 7,000 or 8,000 inhabitants, its roads laid out in avenued lines designated numerically, of its busy prosperous factories and mills, and best of all its lovely Oswego Falls—the Falls that for thousands of years had fallen, splashing, dashing, snow-crested, and glorious, tumbling on in a mad ecstacy of delight to meet the more stately quiet of the river "Oswego." Then as I tried to recall the names of some of them "Neahtawanta" recurred to me with its legend of the Red Maiden, who, offered as sacrifice for her people, was hurled over the Falls in a birch-bark canoe, and instead of being engulphed in the mad rush of the waters passed triumphantly over them, and on to the "Little water by the Great waters,' where her spirit still is seen.

### The Patient.

But even as I dreamed, dimly wondering what my case might be, the train rolled silently into the station, and with a sudden jerk of uncoupling carriages, I awoke to the fact that I must speedily descend. A tall, grave, very lean scion of Uncle Sam's stood on the platform. Whether it was a case of affinity or homœopathic predilection on his part I do not know, probably the latter, for he judiciously advanced to take possession of the smallest dose left standing in the station. I tried to smile as he uncoiled, and his soft billy-cock touched the rim of my hat, but the pain in the man's eyes was so evident that without any preamble I said: "You are Mr. M., and have come to meet me, I know." You see it is only in artificial life that one needs a half-hour's conversation before even a name is known. In less than five minutes I understood that this man was the uncle of my patient, and that the latter was a little sick baby, so sick that the doctors feared he could not live. The house was a very sad one to enter. Several relatives anxiously scrutinised me, but what are people to a nurse who goes to a patient? and in a moment I stood in the chamber where, on a large bed, lay a tiny atom of suffering humanity. The first whiff of the olfactory nerves revealed the trouble; it was unmistakably typhoid. The age of the babe was 19 months, and I knew that right before me was a very rare case indeed, and one of the biggest of battles to fight. The mother was sure he would scream at the sight of a stranger, but the tiny creature lifted its pitiful eyes, and crept a little nearer to my hands, and I crooned, and baby listened till we were fast friends.

### Ice-packing the Baby.

All that night and all next day unceasingly we worked to maintain a cooler temperature of the small body; but the fire rose and rose, despite ice-coils on the head and ice in close proximity to the body, till ice-pack had to follow each other at intervals of three or four hours. But one cannot continue this heroic treatment successfully with the delicate organism of a babe. Coma-vigils, facial contortions, muscular twitchings, increased our care and doubt, and when cyanosis appeared at the extremities I begged that no more ice-pack should be given; but the relief-nurse, mistaking the order, added one more that nearly cost the little life we sought so hard to save. There was no pulsation in the right artery, only the very faintest perceptible in the left, when the mischief was done, and the tiny blue face and collapsed form very slowly responded to the second dose of Spts-vin. Galli. given. Hot water bottles also assisted circulation, till the doctor awakened from a sound sleep and, sure that baby was dead, came and found him returning to life.

### Danger not Yet Over.

After that we gave the patient "ice-rubs," first drenching the body with alcohol to numb the sensibilities of the skin, and found equally good results with less danger from shock. As hyperpyrexia lessened, tympanites became pronounced, and barley-water was substituted for milk. We grew a little hopeful as the fever bridge was passed, but on the day the martyred President was laid to rest, whilst from every house the Stars and Stripes hung listlessly, and in every window was revealed a draped picture of the genial face no more to be seen, whilst the volleys echoed from every State and city and town and village as a protest to Heaven for avenge of the foul deed done, the baby's life drifted again. There had been suspicious symptoms two nights in succession, but when the dreaded hæmorrhage appeared our hearts were verily craven. One could almost feel the touch of the wings of the Angel of Death as he waited for "our baby." The mother could bear no more. She gave her little one back to the God who gave it, and He who heard asked no greater sacrifice than that of submission, for a few days later the mother, father, and uncle came and leaned over the foot of the bed with eyes all wet with happy tears, and baby lifted one wee forefinger whispering to each, "Ma-ma,' "Un-kee," "Dere de Pop-pa." And if you think they were happier than I—well you don't know much about the joy of the snatching of the brand, etc.

### Baby's First Smile.

The great day—the day of baby's first smile and the second of his normal temperature—the long-limbed Uncle took me for a drive. So my wish came to pass, and I saw the Falls as they tumbled and plunged from their terraces above; and we drove along the Oswego River, and looked over the Lake Neahtawanta. As we gazed over the river, with the sunlight glorifying its reflections, it was easy from memory to people this historic place. First a thin line of Jesuit missionaries rose before me; then a more stolid, solid mass of Hollandaise; then it seemed I heard a little stir and shout from British throats, and then a wild grappling of French and English and Dutch, and the leaping of the red man and the hate of race. But my companion touched me and looked at me wonderingly. "Your thoughts are far away," he said, pointing outwards. "Look! yonder is Pathfinder Island. I have a home there, and I will take you to it some day and show you where Fennimore Cooper wrote the story that bears the name of the isle." Yes, perhaps some day; but now the sun is slipping fast behind the gates of the west, and so the horses' heads are turned. One backward look, and I saw it all in the peaceful evening stillness—the river, the lakes, the isles, and the beautiful verdant banks climbing upward from the blue of the water. It was only when the baby called out welcomingly and joyously "Na-na, my na-na," I knew that He had other things for me to do than dreaming.

### Nurses for the Concentration Camps.

We are officially informed by the Secretary of State for the Colonies that a number of additional nurses have been selected for service in the Orange River Colony Colonisation Camps.

The following sail on Saturday in the *Canada*, Miss R. L Massey in charge:—The Misses Jessie Allan, M. W. Aldridge B. G. Alexander, M. Chandler, Ethel Collins, E. M. Clark, Jessie H. Congleton, Isabel Dodgson, A. Fletcher, Elizabeth Fletcher, Agnes Hill, Florence Hird, T. Howard, L. Jessop, A. G. Lean, Joan McLennan, C. M. Phillips, L. E. Strickland, and E. Summerskill.

The following sail on Monday in the *Dunera*, Miss Harriet Green in charge:—The Misses Laura E. M. Baker and Jessie Brown, Mrs. Greig, Misses Barbara Hunter, M. L. Hunter, Christina M. Mackenzie, Rachel McNab, Isabella Macpherson, Isabella Macgillivray, Henrietta Priest, E. Pedlar, J. Ritchie, A. M. Shavrock, H. M. C. Schiemann, Janet Somerville, M. T. Thomlinner, Edith Wingrove, Jessie Ann Wood, and Mrs. Williamson.

Miss Rosa Leake Massey, matron in charge of the first detachment, was trained at the Southern Hospital for Women and Children, Manchester, and at the London Hospital. Since September, 1893, she has been on the private staff of the latter.

Miss Annie Gertrude Lean was trained at Leeds General Infirmary, where she was afterwards sister, and also at Queen Victoria's Jubilee Institute as district nurse. Since December, 1896, she has been working near Manchester.

Miss Constance Margaret Phillips was trained at Leeds General Infirmary, where she was afterwards sister. She has since been doing private nursing.

Miss Harriet Green, matron of the second detachment, was trained at Guy's Hospital, where she was afterwards staff nurse. She has since been sister and assistant matron at the Children's Hospital, Glasgow; matron at the Convalescent Home, Eaglesham; and matron of the Children's Hospital, Bradford. Since October, 1895, she has been doing private nursing as a member of the Nurses' Co-operation.

Miss Laura E. M. Baker was trained at St. John's House, Charing Cross Hospital; has been staff nurse at the Metropolitan Hospital, London, North-Eastern Fever Hospital, and University College Hospital. She has also done private nursing since May, 1898, and is now attached to the Nurses' Co-operation.

Miss Barbara Hunter was trained at the General Infirmary, Leeds, was attached to the Home Hospital Association from 1892 to 1896, and has since been a member of the Nurses' Co-operation.

Miss Isabella Macgillivray was trained at Westminster Hospital and the Royal Infirmary, Edinburgh. She has been private nurse since 1888.

Miss Jessie Ritchie was trained at the Royal Infirmary, Dundee, where she was afterwards staff nurse. Since May, 1897, she has been attached to the Nurses' Co-operation.

Miss Jessie Ann Wood was trained at the Royal Free Hospital and the North West London Hospital. She has since been private nurse and Queen's district nurse in Glasgow.

Mrs. Cecilia Williamson was trained at Glasgow Royal Infirmary, and has since been attached to the Royal Scottish Nursing Association.

Miss Sharrock goes from the Orthopædic Hospital, Great Portland Street, London; Miss Priest, from the City Hospital, Edinburgh; Miss Collins, from the Seamen's Hospital, Greenwich; Miss McLennan, from Towns Hospital, Glasgow; and Miss Summerskill, from the Royal Chest Hospital, City Road, London.

### Appointments.

Cardiff Infirmary.—Miss Mary Smith has been appointed ward sister. She was trained at Cardiff Infirmary for three years and has since been staff nurse for one year.

Cottage Hospital, Swaffham.—Miss Edith B. Bunting has been appointed matron. She was trained at Charing Cross Hospital, and has since been nurse and matron at Iver Langley and Denham Cottage Hospital.

Denes Fever Hospital, South Shields.—Miss Annie Adair and Miss Helen A. Munro have been appointed charge nurses. Miss Adair was trained at Combination Fever Hospital, Govan, and has since been charge nurse for 14 months in the scarlet fever wards at Greenock Infirmary Miss Munro was trained at Knightswood Hospital, Glasgow, where she has since been assistant nurse.

Drumcondra Hospital, Dublin.—Miss Janet Elliott has been appointed lady superintendent. She was trained at the General Infirmary, Paisley, and at Sunderland Infirmary. She has since been charge nurse at the National Hospital, Queen Square, London, district nurse in Perthshire, and lately lady superintendent at Claremont Street Hospital, Belfast.

Eaton District Nurse.—Miss Amy Prowde Nightingale has been appointed district nurse and midwife on the Duke of Westminster's estate at Eaton. She was trained for three years at Bolton Infirmary and Dispensary. She has since been charge nurse under the Metropolitan Asylums Board, and has done private nursing. She holds the L.O.S. certificate.

Farnham Isolation Hospital.—Miss Ellen Howitt has been appointed matron.

Halifax Poor Law Hospital.—Miss Louisa Smithers has been appointed night superintendent. She was trained at Poplar and Stepney Sick Asylum. She has since been sister at Leeds Union Infirmary, superintendent nurse and deputy-matron at the Blackwall branch of the Poplar and Stepney Sick Asylum, and has also held an appointment at Nice Nursing Institute.

Isolation Hospital, Kettering.—Miss Ethel Hope has been appointed matron. She was trained at Burton-on-Trent General Infirmary, and for the last two and a half years has been working in connection with the Northampton Association of Trained Nurses.

Isolation Hospital, Eton Union, Slough.—Miss E. Griffiths has been appointed charge nurse. She was trained at the Seamen's Hospital, Greenwich, and was subsequently nurse for six years. She was then for three years nurse at the South Eastern Fever Hospital, Old Kent Road. She has since been doing private nursing.

Nantwich Union Hospital.—Miss Maud Mellor has been appointed charge nurse. She was trained at Monsall Fever Hospital, Manchester, and has since been assistant nurse at Leeds City Fever Hospital, superintendent nurse at Wolstanton and Burslem Union Infirmary, and nurse at Ruchill Hospital, Glasgow.

Poole Union Infirmary.—Miss Ethel Baker has been appointed superintendent nurse. She was trained at Bristol Union Infirmary, and has since been superintendent nurse at Keynsham Union Infirmary.

St. Albans Union.—Miss Emma Clark has been appointed assistant nurse. She was trained by the Meath Workhouse Nursing Association at St. Joseph's Hospital, Chiswick.

St. Martin's Home for Crippled Boys, Surbiton.—Miss Frederica E. C. Roberts has been appointed lady superintendent. She was trained, for three years, at the Metropolitan Hospital, London, and has since done three years and a half private nursing on the staff of St. John's Home, Norfolk Street, Strand, and nearly two years' district nursing in Southwark.

Southwark Infirmary, East Dulwich.—Miss Frances C. Walker has been appointed night superintendent. She was trained, for three years, at the Metropolitan Hospital, London, and has been ward sister at Southwark Infirmary for twelve months. Miss Walker holds the L.O.S. certificate.

Thornton Joint Hospital.—Miss Elizabeth Mackenzie has been appointed nurse. She was trained at Leith Hospital and the City of Glasgow Fever Hospital. She has since been Queen's nurse and maternity nurse at Barnhill Hospital, Glasgow. Miss Mackenzie holds the maternity certificate of the Glasgow Maternity Hospital.

### Sheppey Workhouse Infirmary.

AN INTERESTING POINT.

The Isle of Sheppey Guardians, at their last meeting, had a letter before them for consideration from Mrs. M. M. Hearn, one of the nurses, complaining that on Thursday January 9th, she refused the matron to attend in any way to the casuals or those admitted otherwise than into the hospital, and was consequently reported to the doctor.

Mrs. Hearn explained that during the last fortnight or more she had had to do double duty owing to the head nurse being away ill, and that it was impossible to take on other work without neglecting the hospital and damaging her reputation as a nurse. She had objected to do the work ever since she came, not that it was objectionable in itself, but because her infirmary duties were standing still while receiving females into the house, and had to be done outside her fixed hours. She was given to understand that on resuming duty Miss Clark, the head nurse, reported that the hospital work had been neglected. Mrs. Hearn, after expressing her desire for a general inquiry to be held into the matter, added that when she brought the question of outside work before the Board some time ago, she received a letter informing her that other arrangements would shortly be made. The work, however, had still to be done by the nurses. There certainly seemed some contradiction somewhere, as the new printed rules agreed upon some time ago to remove other points of friction made no alteration of the old system. There were several other matters which ought to be brought before the Board to make the work of the nurses easier.

The Master said that if Mrs. Hearn were to be relieved of the duties the work must fall upon the head nurse according to the new regulations just printed and exhibited in the rooms, which the matron in giving the order was merely enforcing. The nurses had to admit all females into the house. This had always been the rule at the Sheppey Workhouse, and the Poor Law Board insisted upon it.

Mr. J. R. Brett (the Chairman) was sorry the dispute had arisen, but under the new arrangements about to be made the nurses would be relieved of those duties.

The Master failed to see how the Board could do it without having a big staff of female officers.

The Clerk said that the doctor had written stating that it was not the work of the nurses and that he had always taken that view of the question.

The Master said that the doctor must have seen the rules, where it was distinctly set out to be the duty of the nurses.

Mr. C. Ingleton said that he had a scheme to bring before them in committee which he thought would work well, if adopted, and do away with any future friction on those matters. They would never get competent nurses to do the outside work spoken of. It had been done, but British nurses would not do it to-day—it was not nursing. Things were very different now from what they were ten years ago.

A special meeting of the Board is to be held next Tuesday.

### Novelties for Nurses.

By Our Shopping Correspondent.

IMPROVED "PRINCESS CHRISTIAN" BED-REST.

This simple bed-rest is the patent of a member of the Royal British Nurses' Association, who has obtained special permission to name it after H.R.H. Princess Christian of Schleswig-Holstein. It is on the same principle as an ordinary "American" or "deck" chair, so far as the back is concerned, but the patient being in bed or on a sofa, no seat is required, and all that is necessary is supplied by a strap, with a cushioned foot-rest, to prevent the occupant from slipping down. The strap can be lengthened or shortened by means of buckles. There are several other advantages. One is its extreme lightness and portability making it especially valuable to nurses engaged in district work or in visiting private patients. Again, by means of an improved system of supports, the framework can be readily adjusted to a lower angle than is possible in some rests of a more elaborate make, while it can also be placed quite upright if desired. Those who have to do with invalids, whether in hospital or in their own homes, will appreciate the advantages of being able to vary the position of the patient to so large an extent. The back is of canvas, so that pillows may be dispensed with, or only one used lengthways —a great advantage in hot climates. The materials used are plain and of a serviceable quality, and a small size is made for children's cots. The bed-rest is supplied only by the patentee, Miss Todd, 31 Grosvenor Terrace, York.

DRESS MATERIALS.

(Dress Goods Supply Association, 22 High Street, Manchester.)

From the Dress Goods Supply Association I have before me a box of patterns of useful dress material, both for uniform and ordinary wear. Among these are some strong double-warp zephyrs, which look as if they would stand considerable knocking about in the ward. They are made in half a dozen different colours, and are very moderate in price, being 7½d. a yard, 30 inches wide. A finer zephyr in fast colours is the same width and just half the price. This also has an equal variety of shade from which to choose, and would be very suitable for "the nurse in hot climates." Strong prints at 4½d., galateas at 5d., and drill at 8½d., complete the list of materials for indoor uniform dresses; and then there are white apron linens in two qualities, one a good deal finer than the other, but both appearing to be strong and durable. For out of doors there are alpacas and serges; these would answer the purpose for summer cloaks also. In ordinary materials there is a variety of choice; the "Beatall" coating is marvellously cheap at 11d., wide width, and there are some new zibelines and homespuns of the dark mixtures so fashionable at present. The blouse flannels and "Woolrino" shrunk flannels, in various colours, should also be inspected by nurses looking through their wardrobes and deciding "what to buy."

### Everybody's Opinion.

[Correspondence on all subjects is invited, but we cannot in any way be responsible for the opinions expressed by our correspondents. No communication can be entertained if the name and address of the correspondent are not given as a guarantee of good faith, but not necessarily for publication. All correspondents should write on one side of the paper only.]

### AN APPOINTMENT.

We have received the following from St. Bartholomew's Hospital: "The matron desires me to inform you that the statement in your issue of January 11th concerning Miss Jessie Jayne is incorrect. Her name is not on the books, which have been carefully searched for the past 14 years.—C. M. Riley."

[The announcement was made as it was sent to us officially by the Clerk to the Newcastle Board of Guardians. He informs us that Miss Jayne stated before the Hospital Committee of the Guardians that she was trained "at St. Bartholomew's Hospital." He adds that she "now declines the appointment."—Editor Hospital.]

### SCARCITY OF WORKHOUSE NURSES.

"Rita" writes: In your issue of January 18th there is a letter on this subject by another workhouse nurse. I think her advice to guardians, to look on both sides fairly, very excellent. I have myself been a workhouse nurse for some three years, and I have often noticed how a matron's word will be taken before anyone else's, and even proof of a nurse's innocence refused. This, to say the least of it, is unjust, and is often the means of spoiling many of the best years of a nurse's life. One case came very recently under my notice. A nurse had a patient die very suddenly, and although the doctor stated that there was no neglect whatever on the nurse's part, the matron insisted that the woman's death was caused by carelessness and neglect, and had the matter placed before the committee, with the result that the nurse was called upon to resign. Does not this one case, out of many which might be stated, show that it would be far more satisfactory if Boards of Guardians would inquire a little deeper into matters themselves, and not allow such weight to be attached to a matron's statement, especially one who is not a trained matron?

### LECTURES TO HEAD SISTERS.

"Nurse P." writes: I should like to thank you for the lectures to head sisters. The longer we nurse, the more we realise how much we have yet to learn. I notice Miss Fox says, "If you have ever lain ill," ah, yes, surely here lies the key to the lack of sympathy, the thoughtlessness, the matter-of-fact way in which so many nurses perform their duty. Suffering, surely, is our best teacher; from that we shall learn better than all else, how doubly sensitive the sick always are. I cannot believe that a nurse who has had pain herself would tell an abdominal section immediately after operation that she "made too much fuss," that "the pain was nothing," or that she was "nervous and put on the pain." A nurse, herself experienced in suffering, would never give a patient a cold wet bed-pan or forget the hot-water bottle, or leave the nourishment standing about in the bedroom, because it was "such a bother to keep going up and down." Yet I can vouch for this sort of thing happening to a case in private nursing, during the first week following a very critical abdominal section to a patient having two hospital nurses. Such nurses are not fair either to the surgeon or to the patient, and bring discredit upon their training school. Much adverse comment was made upon your "Nurse's Never." I can assure you that we older nurses thoroughly appreciated it. We know too well how necessary such reminders are. I remember once saying to a young nurse, "Why did you not call the doctor before?" to be answered with "I was afraid the doctor would think me incompetent." That nurse had evidently much to learn. While no one would wish a nurse to be chicken-hearted, I always maintain that a tender touch, a gentle word, and a quiet sympathetic bearing will do no harm, and may be productive of much good to even the most nervous patient. I entreat my fellow nurses not to under-estimate pain. It is enough to be laid aside, enough to have racking pain and ofttimes sleepless nights without nurses adding thereto by thoughtless speech or noisy manner.

### NURSING IN SMALL UNION INFIRMARIES.

"E. J. B." writes: In view of the great and increasing difficulty, both in procuring and in keeping efficient nurses in the smaller union infirmaries, I think that any suggestion as to ways of meeting the want may be welcomed. We cannot ascribe this difficulty to any one cause. It is partly due to the position the trained nurse holds with regard to the master and matron; but also to the extreme monotony of the work, lying as it does almost entirely among the aged infirm, the mentally deficient, or the hopelessly incurable. The life also has the drawbacks of hospital routine and discipline without the advantages of a resident medical staff, and opportunities for keeping up and increasing scientific professional knowledge; there is, too, the isolation from the life of companionship, which is to be found where there is a large body of nurses. The result is, that nurses who for any length of time work in country infirmaries, feel that they have drifted into the shallows and backwaters of their professional career, and that they will soon be left stranded and forgotten by the onward course of the stream. These sepathe drawbacks affect each nurse in a varying degree, but, combined as they are in most cases, there need be no surprise felt that with so many more congenial careers open to them, so few nurses care to devote their lives to workhouse nursing. And these drawbacks all seem to me to be inevitable in the smaller unions. There must be the one paramount authority in the hands of the master or matron, though the Local Government Board has done its best to limit their power of interfering with the strictly nursing arrangements. The other causes are inherent in the nature of the work. Yet our sick and infirm poor must be tended and efficiently tended, and in these days of devoted self-sacrificing work in all directions, it cannot be impossible to find a solution of the problem. But work so monotonous, so hidden from, and inglorious in the eyes of the world, can only be efficiently and persistently performed when undertaken from the very highest motives. Why should we not recognise this, and make it the basis for attempts at improvement? There are, I feel sure, hundreds of earnest, religious and highly-trained nurses who look around for a sphere for their labours. Once lay the obligation of duty on them and they would never hesitate to undertake, and to carry out the meanest, the most monotonous, and the most isolated of work. This religious motive must, however, be acknowledged; it must be the link binding the workers together, so that they feel that, isolated as they are, in their daily work they are members of a body with the same aims and inspirations. My suggestion, the result of much earnest thought, is, that a religious order should be founded, the avowed object being the tending of the sick aged and infirm poor, primarily in union infirmaries, but also in their own homes, almshouses or homes for the dying. Membership in the order should be confined to nurses possessing the qualifications demanded by the Local Government Board; but it might be advisable to admit untrained women as probationers. The rules should be of the simplest possible character, so that they might include women of all schools of thought, simply sufficient to secure that all members should be animated by the Christian ideal and should obey orders as to where and how long they should carry on their work. This agreement, whether taking the form of vows or not, should only be valid for a definite period, say, three or five years. The mother superior, or constitutional head of the order, who would apportion to each member her sphere of labour, would soon, I think, be looked upon by boards of guardians as an agency from which they would be sure of obtaining efficient, conscientious nurses: she should herself, if possible, be a trained nurse with workhouse experience. Of course, any distinctly religious garb would be a fatal mistake. Details of constitution, such as the financial arrangements, the question of a central home, etc., would easily be worked out if once the main principle were adopted—" the enlistment of a regiment of workers under a common standard for a common object for a definite period to work under orders." It would be a truly patriotic as well as a Christian work, and we have no reason to think that England has exhausted her resources of women ready to devote their powers of will, mind, and body to any task she may demand of them. It is easy to understand how the isolated poor law nurse, though entering on her work with high ideals and motives, may come to feel when hampered and daunted and depressed by the unavoidable and inherent difficulties she is certain to encounter, that, perhaps, she may have mistaken her vocation, and that her labours in another field might be more fruitful, both for herself and others. It is then that the sustaining power of an oath of obedience would be most helpful (we all know the comfort it is to feel that our work is not of our own choosing, that our part is only to do it), and the encouragement great to realise that, though occupying an isolated outpost, she was a member of an organised body, bound by the same regulations, owning a common allegiance, fighting the same enemy. I put forward this suggestion as one contribution towards the solution of this pressing and difficult problem, which cannot, I think, be fully solved by any Local Government Board regulations.

### The Nurses' Bookshelf.

Manuals of Employment for Educated Women.—No. IV. Medicine. By Christabel Coleridge. (Walter Scott. Price 1s., cloth 1s. 6d.).

This little manual is the latest addition to the series being brought out " for the instruction of educated women who from choice or necessity may be contemplating the adoption of a profession as a means of livelihood." It is excellently put together and contains practical information of an invaluable kind, not only in what is necessary to be done, but also in much that it is necessary to leave undone if those seeking instruction are to be successful in the calling they adopt. Above all, the author impresses upon her readers that no amount of self-sacrifice, unless accompanied by a good physique, will carry anyone through even the preliminary training necessary to a qualified professional woman. These manuals have been issued at a very low price " in the hope that they may be widely useful and of service in checking that unfortunate tendency among women which has been noted by all students of the subject, and for which parents at least as much as daughters are responsible, of drifting into a profession without regard to suitability and without any satisfactory training." The present manual treats clearly and concisely of the methods by which medical qualifications can be gained by women, the cost of training at the Universities and colleges open to them, with hints as to the best method of starting in practice. How to become dentists, dispensers, and chemists is also included in the list of occupations possible to the trained woman. Everyone who is meditating the practice of medicine and its branches should read this exhaustive and handy manual.

### For Reading to the Sick.

BE YE ALSO PERFECT.

God knows how far from perfection we are; but we are going on with our training and God will continue the education on the other side of the Veil. Age after age we shall go on knowing God more, and becoming more and more able to " see God " and to love Him. All that is " of the earth earthy " will be gone.—*G. H. Wilkinson, D.D.*

I halt to-day; be love my cheerful crutch,
My feet to plod, some days my wings to soar:
Some day; but Lord, not any day before
Thou call me perfect—having made me such.
This is a day of love, a day of sorrow,
Love tempering sorrow to a sort of bliss;
A day that shortens while we call it long:
A longer day of love will dawn to-morrow,
A longer, brighter, lovelier day than this,
Endless, all love, no sorrow, but a song.

*Christina Rossetti.*

When men are just beginning to see into the great intricacies of God's world—when they are beginning to understand a little what Art and Science really mean—we see them, time after time, hurried away and their life apparently broken short. And yet, surely, it is not so: surely the fundamental laws of science and art—the laws of harmony, for instance, with regard to music—may be understood, in a deeper measure, hereafter. May not the man of science, may not the artist, be allowed to see, hereafter, the great principles upon which Art and Science rest, so as to enter into them, and even practise them, yet more thoroughly and perfectly? In all the " secular " employments and tastes of your life, if they be healthy and pure, there may be, nay, without a doubt there will be, in very truth a continuity.—*Bishop Webb.*

We best glorify Him when we grow most like to Him: and we then act most for His glory, when a true spirit of sanctity, justice, meekness, etc., runs through all our actions when we so live in the world as becomes those that converse with the great Mind and Wisdom of the whole world, with that Almighty Spirit that made, supports, and governs all things, with that Being from whence all good flows, and in which there is no spot, stain, or shadow of evil; and so being captivated and overcome by the sense of the Divine loveliness and goodness, endeavour to be like Him, and conform ourselves, as much as may be, to Him.—*Dr. John Smith.*

The night was dark, the door was shut,
But sweet thoughts of the blessèd Name
Of Him who died on Calvary
Swept through my heart, and then there came
Deep prayers, in His mercy, He
Would cast His white robe over me:
. . . . . . . .
And as I prayed, I was aware
That some great light was risen on me;
And, looking upwards in my prayer,
I saw the door was opened wide,
And One was standing at my side
It thrilled my heart to see.
And so, He took me into rest,
From the dreary street with its shadow dim
To the sweet, sweet rest His children know
While their feet are tarrying still below.—*B. M.*

### Notes and Queries.

**The Editor is always willing to answer in this column, without any fee, all reasonable questions, as soon as possible.**
**But the following rules must be carefully observed :—**
**1. Every communication must be accompanied by the name and address of the writer.**
**2. The question must always bear upon nursing, directly or indirectly.**
**If an answer is required by letter a fee of half-a-crown must be enclosed with the note containing the inquiry.**

*Maternity.*

(161) I am a certificated midwife, and was engaged to attend a case, in consequence of which I refused two other engagements. When, however, the confinement for which I was waiting took place, an unqualified nurse was called in and I was never sent for. Can I claim my fees, and compensation for loss of time ?—*Nurse J.*

Certainly you can claim your fees under the circumstances stated.

Will the Editor kindly explain what is meant by "monthly nursing," and say what advantage is gained by it.—*M. N.*

Monthly nursing is that branch of nursing devoted to mothers and their babies during the first month of the infant's life. It is very important that the nurse should thoroughly understand her duties to both the mother and the child, and in consequence fully-qualified, capable women command good fees.

Will you kindly let me know the cheapest—if possible free—home or institution where I could train as midwife ?—*L. B. C.*

The British Lying-in Hospital, Endell Street, St. Giles, W.C. ; the City of London Lying-in Hospital, City Road, E.C. ; the East End Mothers' Home, 394 and 396 Commercial Road, E., are amongst the cheapest. See our advertisement columns for private institutions. There is no free training.

1. Will you kindly tell me if free training is given in maternity nursing at the City of London Lying-in Hospital. 2. How long is the training ? 3. And is free training given at any other hospital in London ?—*E. W.*

1. There is no free training given at this hospital. 2. Six weeks for monthly nurses and three months for pupil midwives. 3. No.

*Gynæcological Nursing.*

(162) In the *Nursing Section* of THE HOSPITAL for January 4th there is a "Lecture on Gynæcology for Nurses," in which the lecturer ends by saying that a nurse "must exercise the greatest care not to contaminate her hands, which she should wash and sterilise." I should like to know how this can be done with the least pain and inconvenience.—*An Old Nurse.*

Wash and sterilise your hands exactly as you would do if you were about to dress a patient suffering from a wound.

*Standard Dictionary.*

(163) I shall be much obliged if you will kindly tell me if "Quain's Dictionary of Medicine" is still the standard work of its kind ?—*M. B.*

"Quain's Dictionary," of which a new edition has just been published, is certainly a standard work of its kind.

*Prolapsed Bowel.*

(164) I should be glad if you or a nurse could propose some treatment for prolapsed bowel, which is very much congested from constant falling, and causes much trouble.—*Sister.*

You ask a question in regard to the treatment of prolapsed bowel ; but all such questions in regard to the treatment of individual cases should be put to the doctor in attendance. We should not only do no good, but also get in hot water all round if we began to make suggestions in regard to treatment over the heads of the practitioners already in attendance upon patients.

*Flat Feet.*

(165) Will you kindly let me know if you can recommend some socks for flat feet. We would rather have a medical man's patent if possible, for they understand better. We have tried Mr. Pond's, of Norwich ; they are no use, the spring does not go under the foot ?—*Sister A.*

Pond's apparatus referred to does very well in many cases, so also does the plan of always wearing boots sufficiently new and stiff to hold up the instep, but the latter is a somewhat expensive arrangement. By a little care and ingenuity it is generally possible to make for oneself a wedge-shaped pad the shape of what should be the hollow of the foot, and to fix this pad inside the boot. The best materials for the purpose are layers of felt or horsehair firmly packed one above the other into a suitably shaped leather case. It must be remembered, however, that the arch of the foot does not extend right across, and that whatever pad is used it must not extend under the outer side of the foot. Of course, the ordinary heel must be abolished. In a really severe case it is impossible to keep up the foot in its proper position by any amount of padding, and in no case is it well to trust to padding alone ; exercises, friction, massage, and in severe cases, irons being required. But all severe cases are matter for the doctor.

*Maternity Work.*

(166) Kindly tell me how I can make myself known amongst the upper middle class as certificated maternity nurse. I have called on four doctors, and given away a hundred cards without result. Would it be etiquette to advertise ?—*Eta.*

Certainly advertise. It is, we are afraid, the only thing you can do.

*Waiting Probationer.*

(167) Some months ago I was accepted as male probationer, but was told at the time that I must await a vacancy. Should I be doing wrong if I wrote and asked if my application had been overlooked, as I have heard nothing further of the matter ?—*Anxious Probationer.*

Write periodically until you get your appointment.

*Home.*

(168) Can you tell me of a home where an elderly clergyman's daughter of very small means could be received ? She is suffering from softening of the brain.—*W. M. C.*

St. Luke's Hospital for Mental Diseases, Old Street, London, seems most likely to receive this case.

Can you kindly tell me what could be done with a patient of 37 who refuses to get up for days together. One nurse who attended her considered it likely she might commit suicide, but the doctor says there is nothing the matter with her.—*M. E. P.*

If there is nothing the matter with her, do nothing. If there is doubt on this point the friends might arrange for a consultation. But all you can do is to carry out the directions of the doctor in change.

*Boracic Acid.*

(169) Will you kindly tell me the quantity of boracic acid crystals to use to a pint of water in order to make a solution of 1 in 30 ?—*Nursery.*

About two-thirds of an ounce.

*Ceylon.*

(170) 1. Will you kindly tell me to whom I should apply for a post on the staff of a nursing association in Ceylon. 2. Are any nurses required for the Boer prisoners stationed in Ceylon, and to whom should I apply for an appointment ?—*Nurse D. F.*

1. To the Lady Superintendent, the Ceylon Nurses' Association, Colombo. 2. We do not think that any are required.

*Lip Reading.*

(171) Please tell me where I could get any information concerning "Lip reading."—*A. M.*

Apply the Secretary, Training College for Teachers of Deaf and Dumb, 11 Fitzroy Square, W.

*Lessons in Nursing.*

(172) I have a morning engagement as teacher, can you tell me where I could get afternoon lessons in nursing ? I have been through the course arranged by St. John Ambulance.—*S. A. C.*

As long as you continue to teach, you must confine yourself to the study of theoretical nursing only. Inquire at your local polytechnic institution, or at the offices of the Young Women's Christian Association, if there have been any popular courses on home nursing arranged for.

*Maternity Nursing.*

(173) Will you kindly tell me how I can get an appointment as maternity or assistant nurse in South Africa, either in a hospital or under Government officials ?—*Anxious to Know.*

Apply to the Immigration Information Office, 31 Broadway, Westminster, S.W., for particulars as to nursing prospects. You had better not go to South Africa unless you have work or friends to go to.

*Untrained Matron.*

(174) Will you kindly tell me if it is permissible for an untrained person to hold the appointment of matron in an isolation hospital, and also say if she has the power to promote a ward-maid to the position of nurse ?—*M. S.*

Unfortunately, the authorities can do as they think fit.

**Standard Books of Reference.**

"The Nursing Profession : How and Where to Train." 2s. net ; post free 2s. 4d.
"Burdett's Official Nursing Directory." 3s. net ; post free, 3s. 4d.
"Burdett's Hospitals and Charities." 5s.
"The Nurses' Dictionary of Medical Terms." 2s.
"Burdett's Series of Nursing Text-Books." 1s. each.
"A Handbook for Nurses." (Illustrated). 5s.
"Nursing : Its Theory and Practice." New Edition. 3s. 6d.
"Helps in Sickness and to Health." Fifteenth Thousand. 5s.
"The Physiological Feeding of Infants." 1s.
"The Physiological Nursery Chart." 1s. ; post free, 1s. 3d.
"Hospital Expenditure : The Commissariat." 2s. 6d.
All these are published by the SCIENTIFIC PRESS, Ltd., and may be obtained through any bookseller or direct from the publishers 28 and 29 Southampton Street, London, W.C.

### Travel Notes.

By Our Travelling Correspondent.

### XCI.—WINTER DAYS IN ROME.

### The Fountain of Trevi.

We all loudly and emphatically declare that we are in no way superstitious, but I believe that there are very few English visitors to Rome, at least, very few feminine ones, who do not wash their hands in the water of the Trevi fountain to ensure the prediction that all who so do will return one day to Rome. I at any rate was not above such weakness, and three times have I religiously washed my hands under that remarkable mass of sculpture. It is erected against the side of a palazzo at the end of the Via delle Muratte, and, as Hawthorne expresses himself, it seems as if some sculptor of the Bernini School had gone mad in the design His words give an excellent idea of this singular erection and of its immediate neighbourhood.

It is a great palace front with niches and many bas reliefs, out of which looks Agrippa's legendary virgin and several of the allegoric sisterhood; while at the base appears Neptune with his floundering steeds and Tritons blowing their horns about him, and twenty other artificial fantasies, which the calm moonlight soothes into better taste than is native to them. . . . . At the foot of the palatial façade was strewn, with careful art and ordered irregularity, a broad and broken heap of massive rock, looking as if it might have lain there since the deluge. Over a central precipice fell the water in a semi-circular cascade; and from a hundred crevices on all sides snowy jets gushed up, and streams spouted out of the mouths and nostrils of stone monsters and fell in glittering drops; while other rivulets that had run wild came leaping from one rude step to another over stones that were mossy, slimy, and green with sedge, because in a century of their wild play Nature had adopted the Fountain of Trevi, with all its elaborate devices, for her own. Finally the water, tumbling, sparkling, and dashing, with joyous haste and never-ceasing murmur, poured itself into a great marble-brimmed reservoir and filled it with a quivering tide, on which was seen continually a snowy semi-circle of foam from the principal cascade, as well as a multitude of snow points from smaller jets. The basin occupied the whole breadth of the Piazza, whence flights of steps descended to its border. A boat might float and make voyages from one shore to another in this mimic lake.

In the daytime there is hardly a livelier scene in Rome than the neighbourhood of the fountain; for the Piazza is then filled with the stalls of vegetable and fruit dealers, chestnut roasters, cigar vendors, and other people whose petty and wandering traffic is transacted in the open air. It is likewise thronged with idlers, lounging over the iron railing and with forestieri who come hither to see the famous fountain. Here, also, are seen men with buckets, urchins with cans, and maidens (a picture as old as the patriarchal times) bearing their pitchers upon their heads. For the water of Trevi is in request, far and wide, as the most refreshing draught for feverish lips, the pleasantest to mingle with wine, and the wholesomest to drink, in its native purity, that can anywhere be found . . .

This vivid account of the fountain occurs in "Transformation," when Miriam encounters the fatal model, who looks over her shoulder as she stares into the water. The book should never be left at home when we start for Rome, for it is an admirable guide-book, and gives such a life-like and fascinating picture of Roman life and character. There are many fountains in Rome: after that of Trevi, the most beautiful are the Fontana del Tritone, in the Piazza Barberini, and the Fontana delle Tartarughe—"Tartarughe" is Italian for tortoises—and it is the prettiest thing imaginable, composed of boys, dolphins, and tortoises. It was certainly partly the work of Taddeo Lantini, a Florentine, and was erected in 1585.

### The Castle of St. Angelo.

What detestable profanation are the so-called civic improvements all over Rome, but more especially in the region round about the Castle of St. Angelo! The magnificent bridge first erected by Hadrian as a splendid approach to his tomb, A.D. 136, became encrusted with the noble works of successive generations; over this ancient roadway what footsteps have passed! The unfortunate Beatrice Cenci and her family; Benvenuto Cellini, gay ruffler as he was, was imprisoned at the fortress and must have crossed the bridge to reach it; whilst his brother Cecchino fell mortally wounded in a street brawl on the middle portion; fighting seemed to run in the Cellini blood, and Benvenuto describes the sanguinary struggles and even murders in which he was himself engaged with extraordinary relish. Near to the Ponte San Angelo is a hideous suspension bridge erected temporarily whilst the unneeded alterations were being made in the original structure and allowed to remain permanently. The tram lines cross it now. On the occasion of my first visit we were handed over to the charge of a *borghese*, as the military porter called him, that is to say, to a civilian. He was an old man, energetic and voluble, but I think some of his histories would not bear too close an investigation. He showed us the passage from the Vatican called *Il corridojo di Castello*, intended to insure the safety of the Popes should flight be necessary from the Vatican; it consists of two passages, one over the other, and the keys were always kept by his Holiness. Hadrian's tomb, which forms the centre round which the Castello was built, was once richly covered with marbles, but these were removed by one of the Popes to make a flooring to a church. We were shown the Cenci cells, but as I said before, it is a questio n whether they ever occupied them, the weight of evidence goes to show that their prison was the Palazzo Savelli (see my paper for March 30th). The cell shown as hers in San Angelo is very small, and has a bedstead composed of three poles driven into the wall on which some rough kind of mattress could rest. Our cicerone, who had I fancy been refreshing himself sufficiently, almost wept in recounting the hapless girl's story, and solemnly assured us that he went every week to the Palazzo Barberini to kiss her portrait; but this I should say was an idea born of a copious midday meal. We crawled into the dungeons and heard many times over how these pertained to "Lucrezia, Giacomo e Bernardino—tre!" each represented by a finger. From these we passed to Cellini's cell, and heard how he had been incarcerated for killing some one and running off with another man's wife—quite venial errors our conductor seemed to think.

From this prison Cellini escaped, but broke his leg in his descent. On the wall is shown a sketch in charcoal fondly stated to be by that gifted swashbuckler, but as charcoal is of all drawing vehicles the most evanescent, we must regard the fact as problematic.

The castle is full of interest, and you will probably visit it more than once.

### TRAVEL NOTES AND QUERIES.

Malaga in the Winter and Spring (Sunflower).—You will find all necessary information in an article of mine for April 28th, 1900. You will see there are several different routes by which you can go. The quickest is via Paris and Madrid.

Rouen in the Summer (Cricket).—I do not think you would find it unpleasantly hot in summer, and the river is always delightful and excursions on it most cheap and easy. As for smells what continental town is free from them, but Rouen is not specially bad, and is a very clean and airy town. Naturally, if you sit about sketching the old quarters, you do sniff a few objectionable odours, but they do no harm I assure you. Try the Hotel du Dauphin et d'Espagne, 91 Rue Jeanne d'Arc; by arrangement, I think, they they would take you for 6 francs per day, or still more reasonable, Hotel Victoria, Rue Verte.

## Figures and Tables

**Figure f1:**